# Correlation Between the Hepatitis C Virus NS3 Protein and CD30 Expression in Diffuse Large B-cell Lymphoma

**DOI:** 10.7759/cureus.65108

**Published:** 2024-07-22

**Authors:** Lili A Saputra, Indrawati Indrawati, Mardiah S Hardianti, Nungki Anggorowati

**Affiliations:** 1 Department of Anatomical Pathology, Faculty of Medicine, Public Health, and Nursing, Universitas Gadjah Mada/Dr. Sardjito General Hospital, Yogyakarta, IDN; 2 Department of Anatomical Pathology, Faculty of Medicine, Universitas Kristen Duta Wacana, Yogyakarta, IDN; 3 Department of Anatomical Pathology, Faculty of Medicine, Public Health, and Nursing, Universitas Gadjah Mada, Yogyakarta, IDN; 4 Division of Hematology and Medical Oncology, Department of Internal Medicine, Faculty of Medicine, Public Health, and Nursing, Universitas Gadjah Mada/Dr. Sardjito General Hospital, Yogyakarta, IDN

**Keywords:** immunohistochemistry, hepatitis c, hcv ns3, cd30, dlbcl

## Abstract

Introduction: One of the etiologies of non-Hodgkin lymphoma (NHL) is chronic infection related to lymphoma pathogenesis, with a high prevalence of hepatitis C virus (HCV) infection seen. In determining the treatment and prognosis of NHL, cluster of differentiation 30 (CD30) immunohistochemical staining plays an important role. High levels of CD30 are found in patients with HCV infection. This study aimed to determine the prevalence of CD30 and HCV expression and its correlation with clinicopathological characteristics of Indonesian diffuse large B-cell lymphoma (DLBCL) patients.

Methods: A total of 86 formalin-fixed paraffin-embedded (FFPE) samples of DLBCL cases were collected over the course of two years from the Anatomical Pathology department at Dr. Sardjito General Hospital in the special region of Yogyakarta, Indonesia. Immunohistochemistry was performed to detect the two markers (CD30 and HCV). Chi-square tests were used to investigate the correlations between CD30 expression and clinicopathological features in DLBCL patients.

Results: The positivity rate of CD30 expression in 86 DLBCL samples was 25.6% (22/86) when using a 0% cut-off, and 7.0% (6/86) while using a 20% cutoff. The positivity rate of HCV expression in DLBCL samples was 34.9% (30/86). Positive CD30 expression, HCV expression and clinicopathological features (age, sex, Ann Arbor stage, extranodal involvement, and morphological variations) did not have statistically significant relationships (p>0.05).

Conclusion: There was no statistically significant correlation between CD30 immunoreactivity (cut-off >0% or >20%) and HCV NS3 expression and clinicopathological features (age, sex, Ann Arbor stage, extranodal involvement, lactate dehydrogenase, Eastern Cooperative Oncology Group status and morphological variants) in DLBCL.

## Introduction

Diffuse large B-cell lymphoma (DLBCL) is the most common subtype of non-Hodgkin lymphoma (NHL) accounting for about 30%-40% of cases globally with a high percentage seen in developing countries [1]. The epidemiology of NHL cases in Indonesia shows that DLBCL is the most common histological type (68.2%) of all B-cell NHL [2]. One of the etiologies of lymphoma is chronic infection related to lymphoma pathogenesis, namely, *Helicobacter pylori* infection, Epstein-Barr virus (EBV), human T lymphotropic virus type 1 (HTLV-1), and hepatitis C virus (HCV) [3].

HCV infection is the leading cause of chronic liver disease worldwide, which is a global health problem. Among the various extra-hepatic manifestations, chronic HCV infection has been associated with various lymphoproliferative disorders. The ANRS (Agence Nationale de Recherches sur le Sida et les hépatites virales) Lympho-C study, which was a large prospective study, confirmed that HCV infection can be associated with B-cell NHL, particularly marginal zone lymphoma (MZL) (39%) and DLBCL (39%). The ability of HCV antiviral therapy to induce a hematological response in HCV-positive NHL patients suggests an etiologic correlation between HCV infection and NHL [4].

While determining the treatment and prognosis of NHL, a recent study showed that cluster of differentiation 30 (CD30) immunohistochemical staining plays an important role. CD30, from the tumor necrosis factor (TNF) receptor superfamily, is expressed on lymphoma cells with minimal cross-reactivity in normal tissues, making it an ideal therapeutic target. CD30 positivity has been reported to have a better prognosis compared to CD30 negativity in non-Hodgkin lymphoma and anaplastic large cell lymphoma [5]. According to the literature, it is stated that high levels of CD30 are found in patients with infectious mononucleosis, hepatitis B virus (HBV), hepatitis C virus, and human immunodeficiency virus (HIV) [6].

To our knowledge, there has been no study that discusses the correlation between HCV infection and CD30 expression in DLBCL. This study aims to examine the correlation between HCV infection and CD30 expression among our local DLBCL samples using the immunohistochemistry (IHC) method and their further correlation with the clinical findings.

This article was part of thesis and previously presented at the Faculty of Medicine, Public Health, and Nursing, Universitas Gadjah Mada (UGM) on May 25, 2022.

## Materials and methods

Samples and data collection

This was a retrospective cross-sectional study involving 86 NHL patients from January 2019 to December 2020 who were diagnosed with DLBCL through a histopathological examination and immunohistochemical examination. DLBCL cases with positive CD20 IHC staining were included. Cases for which paraffin blocks were missing or inadequate were excluded from the study. The tissue blocks were collected from the Department of Anatomical Pathology, Dr. Sardjito General Hospital, Universitas Gadjah Mada, Yogyakarta, Indonesia. Clinical parameters of the patients were obtained from medical records. The study was approved by the Ethics Committee of the Faculty of Medicine, Public Health, and Nursing, Universitas Gadjah Mada (KE/FK/0937/EC/2021).

Clinical characteristics

Clinical data concerning age, sex, Ann Arbor stage, extranodal involvement, Eastern Cooperative Oncology Group (ECOG) performance status and laboratory data, such as serum lactate dehydrogenase (LDH), were extracted from the medical records. The Ann Arbor staging system was divided into early (I/II) and late (III/IV) stages. The ECOG performance status was divided into good (0-1) and poor (2-5).

Immunohistochemical staining

Samples (from 86 DLBCL cases) stained with hematoxylin and eosin (H&E) were evaluated microscopically and subdivided into different morphological variants. Immunohistochemical stains were performed on 4-μm formalin-fixed paraffin-embedded (FFPE) tissue sections according to standard antibody-specific protocols. Antigen retrieval was performed using the Starr Trek detection kit (Biocare Medical, Pacheco, CA). Samples were stained using monoclonal anti-CD30 antibodies (mouse monoclonal antibody MoAb CD30 cell marque Ber-H2) to identify CD30 immunoreactivity. Tissue lymph nodes with staining with the CD30 antibody in Hodgkin lymphoma (HL) cases were used as positive controls. We used IHC to detect the HCV nonstructural 3 (NS3) protein using the anti-hepatitis C virus NS3 protein antibody (rabbit polyclonal antibody; GeneTex, Irvine, CA). In this study, liver biopsies of patients HCV positive on the serologic examination were used as controls.

Analysis of immunohistochemistry staining

The expression of CD30 was measured using a cut-off of 0% and 20%, which has previously been utilized in various investigations with varying staining intensities expressed in percent units. CD30 staining patterns are often homogeneous and strongly membranous and/or paranuclear or Golgi-like [7]. In the cytoplasm of lymphoma cells, NS3 expression could be detected using three different staining patterns: strongly positive, weakly positive, and negative [8]. If more than 30% of the neoplastic cells in any case were positive, the case was declared positive [9].

Statistical analysis

Chi-square tests were used to investigate correlations between HCV NS3, CD30 immunoreactivity and clinicopathological variables such as age, sex, Ann Arbor stage, extranodal involvement, LDH, ECOG status and morphological variations in DLBCL patients. If the p-value was <0.05, statistical relationships were considered significant. IBM SPSS Statistics, version 25 (IBM Corp., Armonk, NY) was used for statistical analysis.

## Results

Clinical data

Male patients accounted for 40 (46.6%) of the 86 DLBCL cases studied, while female patients accounted for 46 (53.4%). There were 35 (40.7%) patients under the age of 60 and 51 (59.3%) patients aged above 60. According to Ann Arbor stage data, 44 patients (51.1%) were in the early stages (stages I-II) and 42 (48.9%) were in the late stages (stages III-IV). There were 35 (71.4%) patients with less than two sites of extranodal involvement and 14 (28.6%) individuals with two or more extranodal involvement sites. Many individuals did not have their LDH levels checked at their first visit, and 31 patients (65.9%) were found to have an elevated LDH level. From 86 cases, only 55 had the ECOG status recorded and a majority had a good ECOG performance status (65.4%); a poor score was exhibited by 34.6%. In 76 patients (88.3%), centroblastic morphological variants were found; immunoblastic variants were found in nine patients (11.6%) and anaplastic variants were found in one patient (0.1%). Table 1 shows the baseline characteristics of the subjects.

**Table 1:** Clinicopathological features of the 86 DLBCL study subjects DLBCL, diffuse large B-cell lymphoma; LDH, lactate dehydrogenase; ECOG, Eastern Cooperative Oncology Group

Clinical characteristics	Overall (n=86), n (%)
Sex	
Female	46 (53.4)
Male	40 (46.6)
Age	
<60 years	35 (40.7)
≥60 years	51 (59.3)
Ann Arbor stage	
I-II	44 (51.1)
III-IV	42 (48.9)
Extranodal involvement site (n=49)	
<2	35 (71.4)
≥2	14 (28.6)
LDH (n=47)	
Normal	16 (34.1)
Elevated	31 (65.9)
ECOG status (n=55)	
Good (0-1)	36 (65.4)
Poor (2-5)	19 (34.6)
Morphology variant	
Centroblastic	76 (88.3)
Immunoblastic	9 (11.6)
Anaplastic	1 (0.1)

Correlation between CD30 expression and clinicopathological characteristics

**Figure 1:**
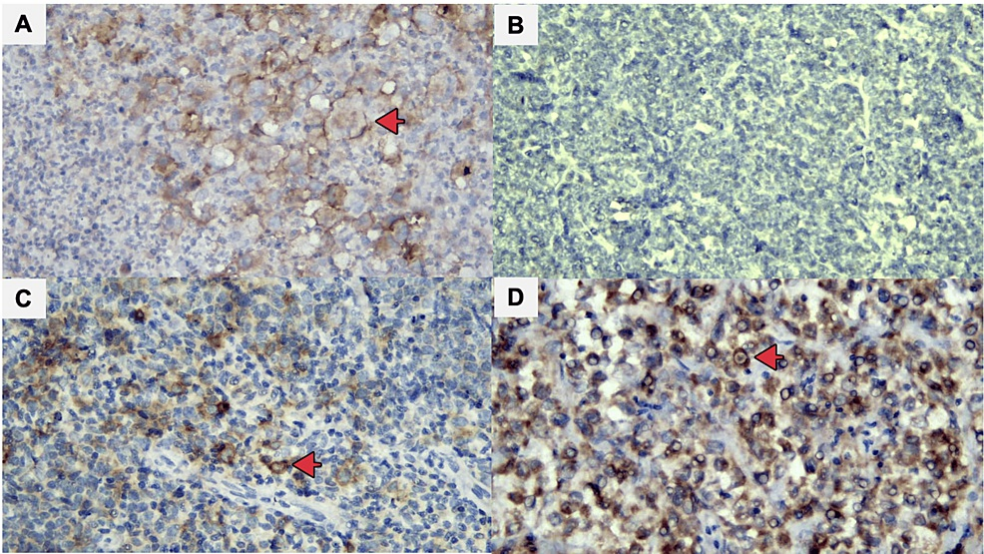
CD30 expression: positive controls for CD30 staining (A), negative CD30 expression (B), positive CD30 expression using a cut-off of >0% (C), and positive CD30 expression using a cut-off of >20% (D). Red arrows indicate cells that are positively stained on the cytoplasmic membrane (A-D, x400).

CD30 expression thresholds were determined using positive percentages of >0% and >20% as cut-offs. A total of 64 patients (74.4%) had negative expression and 22 patients (25.6%) had positive expression with a cut-off of >0%; 80 patients (93.0%) had negative and 6 patients (7.0%) had positive expression with a cut-off of >20% (Figures 1b-1d, Table 2). There were no statistically significant correlations between positive CD30 expression (cut-offs >0% and >20%) and clinicopathological features (age, sex, Ann Arbor stage, extranodal involvement, LDH, ECOG status and morphological variants) (p>0.05) (Table 3).

**Table 2:** Immunohistochemical expression in the study subjects HCV, hepatitis C virus

Immunohistochemical expression	N=86	Total, n (%)
CD30 expression (>0%)		
Positive	22	25.6
Negative	64	74.4
CD30 expression (>20%)		
Positive	6	7.0
Negative	80	93.0
HCV NS3		
Strongly positive	16	18.7
Weakly positive	14	16.2
Negative	56	65.1

**Table 3:** CD30-positive (cut-offs >0% and >20%) and CD30-negative DLBCL clinicopathological characteristics DLBCL, diffuse large B-cell lymphoma; LDH, lactate dehydrogenase; ECOG, Eastern Cooperative Oncology Group; PR, prevalence ratio

Clinical characteristics	0% cut-off		PR (95% CI)	p value (<0.05)	>20% cut-off		PR (95% CI)	p-value (<0.05)
	CD30 negative	CD30 positive			CD30 negative	CD30 positive		
	(n=64), n (%)	(n=22), n (%)			(n=80), n (%)	(n=6), n (%)		
Sex								
Female	33 (71.7)	13 (28.3)	1.25 (0.62-2.52)	0.541	44 (95.7)	2 (4.3)	2.3 (0.52-10.11)	0.305
Male	31 (77.5)	9 (22.5)			36 (90.0)	4 (10.0)		
Age								
<60 years	28 (80.0)	7 (20.0)	1.47 (0.69-3.12)	0.326	31 (88.6)	4 (11.4)	2.91 (0.6-12.8)	0.179
≥60 years	36 (70.6)	15 (29.4)			49 (96.1)	2 (3.9)		
Ann Arbor stage								
I-II	33 (75.0)	11 (25.0)	0.95 (0.48-1.88)	0.899	40 (90.9)	4 (9.1)	1.90 (0.43-8.39)	0.431
III-IV	31 (73.8)	11 (26.2)			40 (95.2)	2 (4.8)		
Extranodal involvement site (n=59)								
<2	27 (77.1)	8 (22.9)	1.06 (0.33-3.35)	0.914	35 (100)	0 (0)	N/A	0.022
≥2	11 (78.6)	3 (21.4)			12 (85.7)	2 (14.3)		
LDH (n=47)								
Normal	13 (81.3)	3 (18.8)	1.54 (0.50-4.79)	0.444	16 (100)	0 (0)	N/A	0.299
Elevated	22 (71.0)	9 (29.0)			29 (93.5)	2 (6.5)		
ECOG status (n=55)								
Good (0-1)	27 (75.0)	9 (25.0)	0.79 (0.33-1.84)	0.602	33 (91.7)	3 (8.3)	N/A	0.196
Poor (2-5)	13 (68.4)	6 (31.6)			19 (100)	0 (0)		
Morphology variant								
Centroblastic	56 (73.7)	15 (24.6)	1.31 (0.33-5.18)	0.667	72 (94.7)	4 (5.3)	0.26 (0.05-1.15)	0.085
Others	8 (80.0)	2 (22.2)			8 (80.0)	2 (20.0)		

Correlation between HCV NS3 expression and clinicopathological characteristics

**Figure 2:**
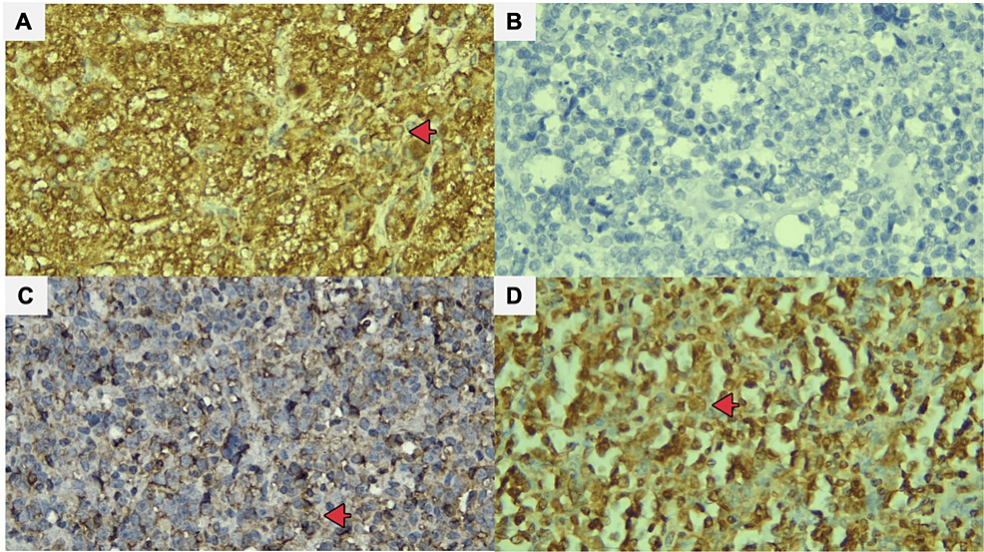
HCV NS3 expression in DLBCL cases: positive controls from liver biopsies (A), negative HCV NS3 expression (B), weakly positive HCV NS3 expression (C), and strongly positive HCV NS3 expression (D). Red arrows indicate cells that are positively stained on the cytoplasmic membrane (A-D, x400).

HCV NS3 expression thresholds were determined using strongly positive, weakly positive, and negative results: 56 patients (65.1%) had negative expression, 16 patients (18.7%) had strongly positive expression and 14 patients (16.2%) had weakly positive expression (Figures 2b-2d, Table 2). There were no statistically significant correlations between positive HCV NS3 expression and clinicopathological features (age, sex, Ann Arbor stage, extranodal involvement, LDH, ECOG status and morphological variations) (p>0.05) (Table 4).

**Table 4:** HCV NS3-positive and HCV NS3-negative DLBCL case clinicopathological characteristics DLBCL, diffuse large B-cell lymphoma; LDH, lactate dehydrogenase; ECOG, Eastern Cooperative Oncology Group; PR, prevalence ratio

Clinical characteristics	HCV		PR (95% CI)	p-value (<0.05)
	Negative	Positive		
	(n=56), n (%)	(n=30), n (%)		
Sex				
Female	29 (63.0)	17 (37.0)	1.13 (0.64-2.01)	0.665
Male	27 (67,5)	13 (32.5)		
Age				
<60 years	25 (71.4)	10 (28.6)	1.37 (0.74-2.54)	0.309
≥60 years	31 (60.8)	20 (39.2)		
Ann Arbor stage				
I-II	31 (70.5)	13 (29.5)	1.36 (0.77-2.42)	0.288
III-IV	25 (59.5)	17 (40.5)		
Extranodal involvement site (n=49)				
<2	18 (51.4)	17 (48.6)	1.7 (0.67-4.31)	0.201
≥2	10 (71.4)	4 (28.6)		
LDH (n=47)				
Normal	10 (62.5)	6 (37.5)	0.60 (0.24-1.45)	0.279
Elevated	24 (77.4)	7 (22.6)		
ECOG status (n=55)				
Good (0-1)	29 (80.6)	7 (19.4)	0.73 (0.28-1.91)	0.557
Poor (2-5)	14 (73.7)	5 (26.3)		
Morphology variant				
Centroblastic	46 (75.4)	15 (24.6)	1.18 (0.39-3.58)	0.730
Others	7 (77.8)	2 (22.2)		

Correlation between HCV NS3 and CD30 expression

In DLBCL cases with positive HCV NS3 expression (strong and weak positive), as many as 30 samples were known to express CD30, with a cut-off of >0% in 8 (26.7%) samples and a cut-off of >20% in 3 (10.0%). DLBCL patients with positive HCV NS3 expression more often showed positive CD30 expression as well, although this result was not significant (Table 5).

**Table 5:** HCV NS3 correlation with CD30 expression HCV, hepatitis C virus; PR, prevalence ratio

Variable	Cut-off >0%		PR (95% Cl)	p value (<0.05)	Cut-off >20%		PR (95% CI)	p-value (<0.05)
	CD30 negative	CD30 positive			CD30 negative	CD30 positive		
	n=64, n (%)	n=22, n (%)			n=80, n (%)	n=6, n (%)		
HCV NS3								
Positive	22 (73.3)	8 (26.7)	1.06 (0.51-2.19)	0.866	27 (90.0)	3 (10.0)	1.5 (0.38-5.87)	0.421
Negative	42 (75.0)	14 (25.0)			53 (94.6)	3 (5.4)		

## Discussion

In this study, it was found that DLBCL cases had an equal sex distribution (46 females, 40 males) in Yogyakarta. This finding is different from other studies that revealed that DLBCL cases are more common in men than women, but is similar to the findings of Morais-Perdigão et al. (21 females, 20 males) and Hardianti et al. (10 females, 10 males) [10,11]. The difference in the incidence of lymphoma against sex is not fully known, but several theories state that hormonal changes in women during pregnancy reduce the risk of lymphoma occurrence [12]. In this study, where DLBCL cases were more common in women, it may be due to the fact that in the sample, 59.3% patients belonged to the age category of ≥60 years, and hence, more women were in the menopause age.

DLBCL is most typically found in people who are in their 70s and our study also found that DLBCL was more prevalent at ≥60 years of age (60%). According to the Ann Arbor clinical stage data, 48.6% patients were at an advanced stage (stages III-IV), while for extranodal involvement sites, there were 12 (29.3%) patients with two or more extranodal involvement sites. For the division of variants based on morphology, the centroblastic variant was seen in 87.1%, immunoblastic in 11.4%, and anaplastic in 1.5%. This is similar to the results from several studies that reported the centroblastic variant as the most common morphological variant discovered in the sample [13].

There have been various studies using cut-offs of >0% and >20%, including a previous research conducted at Dr. Sardjito General Hospital [14]. In this study, DLBCL cases expressing CD30 with a cut-off of >0% and >20% were 25.6% and 7% of the 86 samples, respectively. A previous research conducted at Dr. Sardjito General Hospital found that the results of positive CD30 expression in DLBCL with a cut-off of >0% and >20% were 13.5% and 1.9%, respectively, of 104 samples, while other studies found results of 16% and 6%, respectively, of 50 samples [13]. The above-mentioned values are in accordance with a systematic review of 28 articles that stated that positive CD30 expression in DLBCL varied in the range of 3.5% to 59.1% at a cut-off of >0% and ranged from 2.5% to 36.7% at a cut-off of >20% [15]. The existence of a range of positive values is due to the absence of standard criteria in assessing CD30 expression in DLBCL.

The results of data analysis in this study showed that there was no statistically significant correlation between positive CD30 expression (cut-offs >0% and >20%) and clinicopathological characteristics (p>0.05). Rodrigues-Fernandes et al. stated that CD30 expression with a cut-off of >0% and >20% in DLBCL was significantly associated with the age group of <60 years, poor ECOG performance status, extranodal involvement (two), increased LDH levels and Ann Arbor stages III-IV. This finding contradicts the results of this study where CD30 expression in DLBCL at a cut-off of >0% was found to be highest in the ≥60 years age group and with less than two extranodal involvement sites. However, there are other studies that state that there is no significant difference in age-related CD30 expression when using both cut-off values [16].

The reverse transcriptase-polymerase chain reaction (RT-PCR) technique to detect the presence of RNA virus is the gold standard for supporting investigations to detect HCV; however, this technique is quite expensive and requires facilities that only a few anatomical pathology laboratories have. Meanwhile, the immunohistochemical technique has been used very often by pathologists at a fairly low cost [17].

NS3 is one of the non-structural proteins in HCV and plays a role in the replication process and has a fairly high specificity in detecting HCV by immunohistochemical techniques. A study compared HCV testing using immunohistochemical techniques with RT-PCR. The results showed that the sensitivity and specificity of the immunohistochemical technique were 71% and 88%, respectively [17]. There have been many studies involving HCV testing by detecting the NS3 protein in hepatocytes and lymphoma cells using immunohistochemical techniques. NS3 expression is detected in the cytoplasm of lymphoma cells with three outward patterns, namely, strongly positive, weakly positive, and negative [8]. In this study, it was used as a control in the liver biopsy of patients with HCV positivity on serological examination.

From the results shown in Table 4, it can be deduced that there is no significant relationship between HCV NS3 positive expression and clinicopathologic characteristics. However, the results were consistent with other similar studies, as from 86 patients, there were 30 patients with positive HCV NS3 expression, or as much as 34.9%. This finding is in accordance with the results of another study that found positive expression of HCV NS3 in B-cell lymphoma in as much as 45.94% with the proportion being higher in DLBCL cases than MZL [8]. Likewise, other epidemiological studies state that the percentage of NHL associated with HCV in various countries ranges from 0% to 50% [18].

Based on previous studies, HCV-positive DLBCL has clinicopathological characteristics, which are more common in patients aged ≥60 years, patients at advanced stages, with elevated LDH levels, and high International Prognostic Index scores [19-24]. In this study, similar results were obtained; DLBCL with strongly positive and weakly positive HCV NS3 expressions was mostly found in women (37.0%), in the age group ≥60 years (39.2%), patients with Ann Arbor stages III-IV (40.5%), those with increased LDH levels (22.6%), good ECOG status (19.4%), and centroblastic morphological variants (24.6%).

In this study, HCV NS3 was stained on lymphoid cells by >30% and spread to both lymphoma cells and lymphoid cells. The presence of the HCV NS3 antibody on lymphoid cells indicates the presence of the HCV antigen in DLBCL, which means that there is an HCV infection process that precedes the process of malignant transformation and HCV can play a role in this process.

CD30 protein can be released in a soluble form, also called soluble CD30 (CD30s). Elevated levels of CD30s were found in liver disease with severe HCV infection indicating activation and expansion of Th2 cells, so CD30s levels are useful as markers of chronic HCV infection. In the study by Foschi et al., serum samples were taken from 29 patients and examined for HCV antibodies by the ELISA technique, and for HCV-RNA by the PCR technique. CD30s levels were examined using the ELISA technique. The results showed that nearly half of the patients (16 samples) with chronic HCV infection had elevated CD30s that was associated with disease activity and severity [25].

Reiser et al. investigated CD30 expression in HCV-infected liver samples using immunohistochemical techniques with positive CD30 expression found in 20 of 37 samples (54.05%) [26]. In this study, DLBCL with positive HCV NS3 expression was found in 30 samples that expressed CD30 at cut-offs of >0% and >20% in 8 (26.7%) and 3 (10.0%) samples, respectively, although these results are not statistically significant. In DLBCL cases with positive HCV NS3 and CD30 expressions, it is recommended to give antiviral therapy, either a combination of pegylated interferon-α (Peg-IFN-α) and ribavirin (RBV) or direct-acting antiviral (DAA) therapy. DAA therapy in HCV-positive NHL patients is said to eradicate HCV and regress lymphoma [4].

There are several limitations to this study. One is neither RT-PCR nor serological examination of HCV infection was carried out, so false positive values might be possible. In addition, the examination of HCV infection using immunohistochemical techniques is not a routine examination carried out in anatomic pathology laboratories, so standardisation is still needed in the preanalytical process and staining procedures. Reading immunohistochemical interpretation can also be influenced by the subjectivity of the reader, so in this study, the interpretation was carried out by two pathologists. Data on the clinicopathology of the patients were incomplete because routine LDH examination and assessment of ECOG performance status were not performed.

## Conclusions

The prevalence of DLBCL with positive CD30 expression at the >0% cut-off was 24.2% and at the >20% cut-off was 7.2%; the prevalence of DLBCL with positive HCV NS3 expression was 24.2%, according to our findings. There was no statistically significant correlation between CD30 immunoreactivity (cut-off >0% or >20%) and HCV NS3 expression and clinicopathological features (age, sex, Ann Arbor stage, extranodal involvement, LDH, ECOG status and morphological variants). DLBCL with negative expression of HCV NS3 more frequently exhibited positive CD30 expression as well, although in this study was not statistically significant.
